# Evolution of Cohesion between USA Financial Sector Companies before, during, and Post-Economic Crisis: Complex Networks Approach

**DOI:** 10.3390/e24071005

**Published:** 2022-07-20

**Authors:** Vojin Stević, Marija Rašajski, Marija Mitrović Dankulov

**Affiliations:** 1 University of Belgrade-School of Electrical Engineering, Bulevar Kralja Aleksandra 73, 11120 Belgrade, Serbia; vojin.stevic@gmail.com (V.S.); rasajski@etf.bg.ac.rs (M.R.); 2 Institute of Physics Belgrade, University of Belgrade, Pregrevica 118, 11080 Belgrade, Serbia

**Keywords:** complex networks, time series, economic systems, evolution of community structure

## Abstract

Various mathematical frameworks play an essential role in understanding the economic systems and the emergence of crises in them. Understanding the relation between the structure of connections between the system’s constituents and the emergence of a crisis is of great importance. In this paper, we propose a novel method for the inference of economic systems’ structures based on complex networks theory utilizing the time series of prices. Our network is obtained from the correlation matrix between the time series of companies’ prices by imposing a threshold on the values of the correlation coefficients. The optimal value of the threshold is determined by comparing the spectral properties of the threshold network and the correlation matrix. We analyze the community structure of the obtained networks and the relation between communities’ inter and intra-connectivity as indicators of systemic risk. Our results show how an economic system’s behavior is related to its structure and how the crisis is reflected in changes in the structure. We show how regulation and deregulation affect the structure of the system. We demonstrate that our method can identify high systemic risks and measure the impact of the actions taken to increase the system’s stability.

## 1. Introduction

Economic crises negatively impact people’s lives. They influence every aspect of individual and social development. Therefore, it is essential to prevent a crisis or alleviate its impact by promptly taking appropriate action. Thus, it is necessary to understand the economic system’s functioning and behavior before, after, and during the crisis. Different approaches have been applied towards that end, including economic [1,2] and quantitative approaches [3,4,5,6,7,8,9].

The economic system is a complex system consisting of many interacting units whose collective behavior cannot be inferred from individual units’ behavior. The behavior of the complex system is determined by its structure [10,11]. To understand the behavior and function of a complex system, one needs to describe its structure and understand how this structure evolves. Complex networks theory provides tools for the inference of the structure of a wide range of systems, including biological [12], social [11], technological [13], and economic systems [14]. The construction of economic networks is mostly achieved by mapping the flow of funds between companies [15] or transforming time series into a correlation matrix [14]. These two networks are complementary, although they overlap to a certain extent. The former network requires more time-consuming data collection, while the advantage of obtaining a network from time series is in its simplicity and the availability of data. The appropriate method for efficiently extracting information from time series is essential since it provides insights into the system’s structure at a relatively low data collection cost.

Existing methods for obtaining networks use as input data either the time series of logarithmic returns [16] or methods based on detrended logarithmic returns [14]. Both are derived from the time series of prices. The direct use of the prices is often avoided because they are non-stationary and contain trends.

Current works obtain a network from a correlation matrix by applying filtering methods such as the minimum spanning tree (MST) [17], planar maximally filtered graph (PMFG) [14], and threshold method [18]. Complex networks, including economic networks, are characterized by the rich mesoscopic structure, known as communities [19]. MST and PMFG techniques are not appropriate for analyzing communities in a network and their interconnections as they focus on including all nodes in the network, disregarding stronger intra-community connectivity [20]. Existing methods that use the threshold method do not differentiate between relevant and less relevant edges, filtering out essential information about the system.

The economic crisis is a common research topic [3,4,5,6,7,8,9]. These works contribute to a better understanding of crisis by examining the system’s functioning using different quantitative methodologies. This diverse approach is especially beneficial for a better understanding of the crisis. Input data are the time series of market indices coming from different countries [21] or time series of companies’ stock prices constituting, for example, the S&P 500 index [6]. The former data do not include many constituents and are used in works where the subject understands global interaction. The latter focuses on the USA market, which can have up to 500 constituents, the largest USA companies, and all sectors.

In this work, we propose a new approach for obtaining the network from price time series, which provides insight into the system’s structure. Our motivation is to obtain an optimal network containing sufficient information and as few edges as possible, allowing efficient analysis. Moreover, we want to gain insight into the change in the system’s structure due to the economic crisis. We use a time series of prices and apply detrending to those series. We demonstrate that the system’s structure can be inferred from these data, thus broadening the dataset options for the empirical study of complex systems. We applied this approach for obtaining a network from a correlation matrix that differentiates between edges based on their relevance to network topology. Using this approach, we studied the evolution of the USA financial sector’s network structure. The financial sector is the heart of the economy since companies in this sector enable a flow of funds through the economy. The 2008 economic crisis was catalyzed by subprime mortgage-backed securities in the USA and spread to mutual funds, pensions, and other parts of the financial sector, with national and global impacts. The USA financial sector occupied a central place in the emergence and development of the 2008 crisis. For these reasons, we focus on studying the evolution of company relations in this sector. Our input data present all companies from the domestic financial sector, the source of the 2008 crisis. We identify the relationship between the economic system’s structure and behavior before, during, and after the crisis. Our analysis shows how an economic crisis affects a system’s structure on a mesoscopic level. We examine the relationship between inter- and intra-community connectivity. We show that, using this approach, we can detect crises and different interventions by governments and policy-makers by examining the community structure and their connections. By adding the different perspectives of observing the system’s behavior in crisis, we contribute to a better understanding of connectivity and relations within the economic system.

The rest of the paper is organized as follows. In Section 2, we give an overview of previous work on the following topics: time series processing, obtaining networks from correlation matrix, and methods for studying economic crisis. In Section 3.2, we provide a detailed description of our approach. We present our results in Section 3.1, and discuss these results and conclude in Section 5.

## 2. Related Work

### 2.1. Time Series Processing

The time series of prices are not suitable for calculating the correlation matrix since they have strong trends and are non-stationary. Different methods were applied in order to overcome these problems. One of the methods is based on the simple transformation of prices into logarithmic returns [6,21,22,23,24,25], derived as $r_{t}=\ln\frac{P_{t}}{P_{t-1}}$, where $P_{t}$ presents the price at time *t*. These series fluctuate around the mean, which is constant and close to zero.

The other methods apply detrending techniques on the time series of returns [14,26,27,28]. These detrending techniques differ according to trend calculation. Zhao et al. [14] make a cumulative time series of returns and calculate the trend for each series separately based on the detrending fluctuation analysis technique [29]. Random matrix theory is used to calculate market component, representing the trend in [26,28]. Musmeci et al. [27] calculate the market component based on average returns for all companies considered in the analysis.

Some works used the auto-correlation of time series of returns to derive residuals, which are then used to calculate the correlation matrix [17,30]. Dynamic conditional correlation multivariate generalized autoregressive conditionally heteroscedastic (GARCH) model, DCC-MVGARCH, is used in these works.

### 2.2. Obtaining Network from Correlation Matrix

Obtaining a network from a correlation matrix suitable for gaining insights into the system’s structure is a complex problem. It involves the usage of an appropriate filtering method. The method should ensure that relevant information is present in the network and that redundant edges are removed. Not satisfying any of the two requirements can lead to false conclusions. Existing filtering methods include the minimum-spanning tree (MST) [17,30,31,32], planar maximally filtered graph (PMFG) [6,14,33,34] and threshold method [18,20,21,22,23,28].

The threshold method filters out information based on correlation strength, while MST and PMFG combine correlation strength to include all graph nodes and planarity. From the perspective of inter- and intra-connectivity between communities, inclusion and planarity criteria result in a connected graph at the price of not including all relevant edges. Onnela et al. [20] compared the threshold method with MST and showed that a threshold network with the same number of edges as MST results in a disconnected graph. These results imply that intra-community edges are more robust than edges between the communities. Moreover, in [6], PMFG leads to the conclusion that in times of crisis, communities are less connected than they out of crisis, which is in contrast to results obtained using random matrix theory [3,7].

The threshold method is more suitable for analyzing community structure in the network. However, the problem is finding the optimal threshold value. A lower threshold is desirable to include as much information as possible. On the other hand, a higher threshold is preferable since it provides a sparse network, which is easier for analysis. The optimal threshold is the one that filters out noise from the network structure and leaves the edges that carry relevant information about mutual relations between entities. Onnela et al. [20] proposed clustering coefficient as the criteria for determining the threshold value. However, there is no substantial evidence that the clustering coefficient is more relevant than other network measures.

X. Cao et al. [28] calculated the optimal threshold by comparing clustering coefficients, the average shortest path length, and the size of the giant component between random graph and empirical network for different threshold values. They determine the optimal threshold at which the structural difference between empirical and random networks is at the highest level. While these network properties are one of the most investigated ones, they are not inclusive of other topological properties [35]. The work from C. Orsini et al. [35] indicates that the degree sequence, joint degree matrix, average clustering coefficient, and its dependence on the node degree are sufficient to describe the topology of most of the networks. In contrast, the giant component’s average shortest path length and size depend on these properties.

Xue Guo et al. [18] determine the threshold based on the community’s correlation strength. This approach underestimates the inter-connectivity between communities as higher importance is given to intra-community edges. Inter-community edges impact the diffusion process in a network and should be recognized appropriately. Moreover, according to the max-flow min-cut theorem, edges between communities are essential since the information flow is maximal through them.

S. Kumar et al. [21] set different thresholds to show how network characteristics, such as the component number and maximum clique size, change with the threshold. Xia et al. [23] determine the threshold by using the probability distribution of correlation coefficients and setting the threshold at the expected value plus multiples of standard deviations.

The mentioned threshold methods do not provide quantitative insights into how much information is filtered from the network. The complete correlation matrix carries all information about the structure of the network. Once threshold filtering is applied, a certain amount of information is lost. Therefore, it is essential to have quantitative insight into how much information we included in the network. It is vital to see which edges carry the relevant information about the systems’ topology and which are redundant. Here, we propose a quantitative measure based on the network’s spectral properties to determine the optimal value of the threshold.

### 2.3. Crisis Examined Using Quantitative Methodologies

Different quantitative methods have been applied to better understand the impact of the crisis on the economic system. V. Filimonov et al. [5] used the Poisson Hawkes model and developed a measure to determine whether price fluctuations are due to an endogenous feedback process as opposed to exogenous news. A. M. Petersen et al. [4] studied cascading dynamics and related the Omori, productivity, and Bath laws with financial shocks. G. Oh et al. [36] used entropy density function in return time series, while K. Yim et al. [37] used the Hurst exponent.

Complex networks theory is also used for the analysis of crisis impact. X. Cao et al. [28] have shown that the crisis impacts the average degree, size of the giant component, and clustering coefficient. S. Kumar et al. [21] presented how the crisis affects the formation of clusters and the structure of minimum spanning trees. A. Nobi [24] showed the impact of the crisis on degree distribution and cluster formation. M. Wilinski [38] showed that MST changes structure from a hierarchical scale-free MST to a superstar-like MST decorated by a scale-free hierarchy of trees. L. Zhao et al. [6] examined how the crisis affects the number of communities and inter-sector edges.

Existing methods that use complex networks to analyze the impact of a crisis primarily consider either mapping country indices [21] or the constituents of leading indices S&P 500 [6]. The former network comprises nodes representing different countries, while the latter network nodes represent companies from different sectors. These companies are, for example, for index S&P 500, the largest 500 companies in the USA. This work demonstrates our approach to studying the evolution of relations between companies in the USA financial sector. We show that laws and policies strongly influence the system’s structure. The network’s community structure reflects the pre-crisis, crisis, and post-crisis periods.

## 3. Materials and Methods

### 3.1. Data

Innovative solutions such as derivatives and securitization in the financial sector that were not followed by developing the system’s regulatory framework created a bubble in the housing and credit supply markets. The bubble burst in 2008 due to the subprime mortgage crisis, which led to a worldwide economic crisis. This work studies the long-term relations between companies in the USA’s financial sector and its evolution from 2002 until 2017. This period includes the time before the 2008 crisis, the period during the crisis, and the economic recovery period. The financial sector includes companies whose main economic activity is asset management, real estate investment trusts (REITs), banks, insurance, and municipal funds.

We obtained data from the publicly available Finance Yahoo database https://finance.yahoo.com/ accessed on 27 September 2018 which contains various information data about the company’s values and how they have changed with time. The database comprises different data types, for instance, opening, closing, intraday, adjusted closing prices, and trading volume. The information is given for different aggregation intervals: day, week, and month. For this study, we used adjusted daily closing prices. The closing price means that the price is taken at the end of the business day after trading is closed. The price fluctuates between the opening and closing of a business day. Adjusted means that the price is corrected to exclude the effect of dividend pay-out and stock split. The impact of dividend pay-out and splits of stock would provide misleading information. A split or dividend pay-out can significantly change the price, although the company’s real value did not change.

For each year T∈{2002,…,2017}, we collected the time series $x_{i}^{T}\left(t\right)$ of the adjusted closing price at the end of each trading day *t* for each company *i*. Each time series’ length equals the one-year or 252 trading days. The number of companies in each year $N_{c}\left(T\right)$ varies since some companies were founded after 2002, and some of them were closed before 2017. Table 1 shows the number of companies active in year *T* in the USA financial sector according to the Yahoo Finance database.

Table 1 shows that the number of companies in the USA financial sector grew by 7.5% per year on average before 2007. The crisis and economic recovery period from 2007 until 2015 had much slower growth, with an average relative increase in the number of companies of approximately 2.7%. There was a certain stagnation of this growth in 2016 and 2017.

### 3.2. Methodology

This work proposes a method for determining the network of relations between companies based on their stock price time series. We use this method to study the evolution of cohesion of financial sector companies whose stocks are publicly traded on the USA’s stock exchange. With this method, we explore the evolution of mutual influences and how this evolution is shaped by different critical events, such as the world economic crisis in 2008.

Our method consists of three steps. In the first step, we perform a detrending time series of stock prices for each considered company. In the second step, we calculate the matrix of Pearson correlation coefficients using this detrended time series. In the final step, we apply the threshold filtering of correlation coefficients to extract the companies’ network of relations. We then analyze and compare the topology of the networks obtained for different years.

#### 3.2.1. Time Series of Prices for Obtaining Network

**Figure 1:**
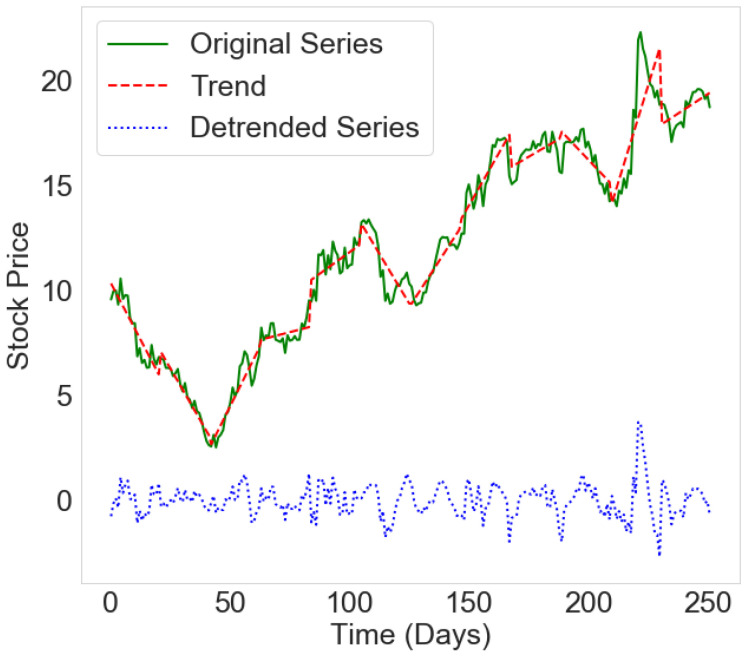
Example of a time series of prices for one company belonging to the USA financial sector. The green line is the original time series, the red line shows the trend, and the blue line is a detrended time series of prices.

In our approach, the companies are represented by nodes, and edges represent companies’ relationships. As input data, we use the time series of stock prices. We consider the time series of each company’s adjusted daily closing prices. The considered time series are non-stationary and have strong trends, as can be seen in Figure 1 (green line), which are often the consequence of different external influences. The non-stationarity of the time series and the trends can lead to false, highly positive, or negative correlations between companies. To avoid this, we remove the trends by detrending the time series using the method proposed in [29]. The detrended time series is the time series of the fluctuations.

Original time series $x_{i}^{T}\left(t\right)$ consists of 252 values of adjusted daily stock prices of the company *i* during year *T*. In [29], the authors considered the differential time series of fluctuations and then performed detrending on the cumulative time series. Our original time series are already cumulative, thus omitting this step in our calculations. We divide the time series on *k* non-overlapping segments of equal size *l*, so that $k=\frac{n}{l}$. We determine the linear trend of time series $x_{i}$ by fitting the equation $y_{i}^{j}\left(t\right)=a_{i}^{j}\times t+b_{i}^{j}$ and determining the coefficients $a_{i}^{j}$ and $b_{i}^{j}$ for each segment *j*, as can be seen in Figure 1 (red line). The detrended time series equals the original time series minus the trend on each segment, i.e., $\bar{x_{i}^{T}}\left(t\right)=x_{i}^{T}\left(t\right)-y_{i}^{j}\left(t\right)$. The resulting time series is stationary, and its average value is approximately zero. By removing the trend typical for period *l*, we only consider fluctuations that result from mutual influence between companies.

We apply detrending to each company’s time series. The detrended time series are used for the calculation of the Pearson correlation coefficient matrix for year *T*, where each element of the matrix is calculated using the following formula:

(1) $${\hat{\rho }}_{i,j}^{T}=\frac{\sum_{t=1}^{n}\left(\bar{x_{i}^{T}}\left(t\right)-{\hat{\mu }}_{\bar{x_{i}^{T}}}\right)\left(\bar{x_{j}^{T}}\left(t\right)-{\hat{\mu }}_{\bar{x_{j}^{T}}}\right)}{\sqrt{\sum_{t=1}^{n}{\left(\bar{x_{i}^{T}}\left(t\right)-{\hat{\mu }}_{\bar{x_{i}^{T}}}\right)}^{2}\sum_{t=1}^{n}{\left(\bar{x_{j}^{T}}\left(t\right)-{\hat{\mu }}_{\bar{x_{j}^{T}}}\right)}^{2}}},$$

where $\bar{x_{i}^{T}}\left(t\right)$ and $\bar{x_{j}^{T}}\left(t\right)$ are the detrended time series of companies *i* and *j* in year *T*, ${\hat{\mu }}_{\bar{x_{i}^{T}}}$ and ${\hat{\mu }}_{\bar{x_{j}^{T}}}$ are estimated average values over period n=252 for the detrended time series of companies *i* and *j*. The matrix with ${\hat{\rho }}_{i,j}^{T}$ elements is symmetrical and takes values from −1 to 1.

In order to obtain the network of mutual influences between considered companies for the year *T*, we only take into account the correlation coefficient with a value above a certain threshold θ, i.e.,

(2) $$w_{i,j}^{T}=\left\{\begin{array}{c} {\hat{\rho }}_{i,j}^{T}\;\mathrm{if}\;\;{\hat{\rho }}_{i,j}^{T}>\theta \\ 0\;\mathrm{if}\;\;{\hat{\rho }}_{i,j}^{T}\leq \theta \end{array}\right..$$

Determining the threshold value θ is not a simple task. In their approach, Živković et al. [39] assumed that the most optimal value of the threshold can be determined from the relation between the threshold value and the size of the largest component in the network obtained for that value. The giant component is the largest set of connected nodes in the network [10]. The dependence of the size of giant component *S* on the threshold value θ has a characteristic steep decline in the giant component’s size for a particular value of the threshold $\theta_{c}$. The abrupt deterioration implies the detachment of a group of nodes forming separate components. $\theta_{c}$ is the threshold value for which one can observe essential changes in the network structure. The threshold value is determined as the one which is slightly smaller than $\theta_{c}$.

**Figure 2:**
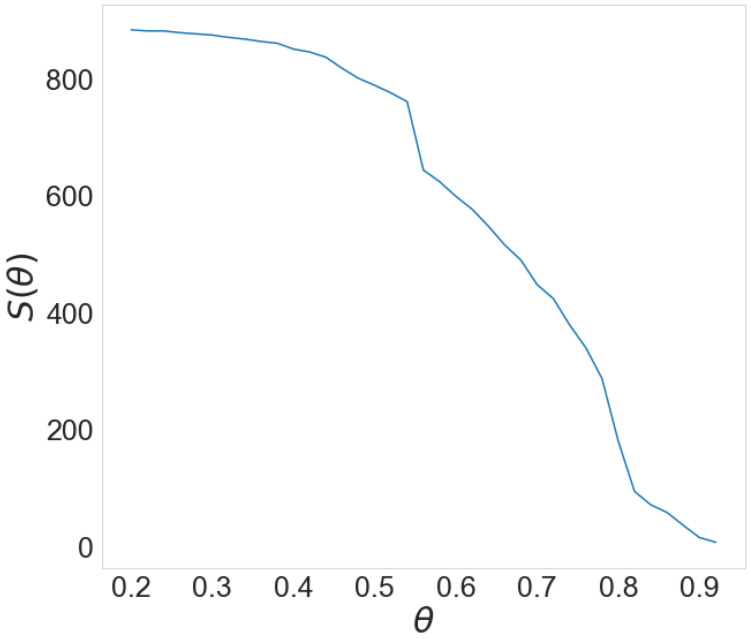
Dependence of size of giant component (S) on value of threshold (θ).

Figure 2 shows the dependence of the size of the giant component *S* on the value of threshold θ for the financial sector in the year 2015. There are two steep drops in the value of the giant component’s size, one for the values of the threshold starting from 0.54 and ending at 0.56, and one starting from 0.78 and ending at 0.82. This indicates that observed networks elapse through a series of significant structural changes; thus, it is hard to determine the optimal threshold value.

For these reasons, we adopted a different approach. We determine the optimal threshold for filtering the correlation matrix based on the networks’ spectral properties. The probability distribution of the eigenvalues of the adjacency matrix fundamentally describes a system and contains the complete information about its topology [40,41,42]. Different networks, such as Erdos–Reniy and Barabasi–Albert graphs, have different probability distributions of eigenvalues. The difference between the two networks is proportional to their structural differences.

We compare the empirical economic network’s spectra with the spectra of different random networks to demonstrate our claims. C. Orsini et al. [35] proposed a method to create a line of random networks, each topologically more similar to an empirical network. We obtained a network from the correlation matrix by applying the threshold method and using it as the empirical network. We generated three random networks (RNs) based on the empirical network properties. RN1 has the same average degree as the empirical network, while other topological properties are random. The RN2 has the same degree sequence and consequently, the average degree as the original network. The RN3 has the same joint degree matrix, degree sequence, average degree, and the most similar topology to the empirical network.

**Figure 3:**
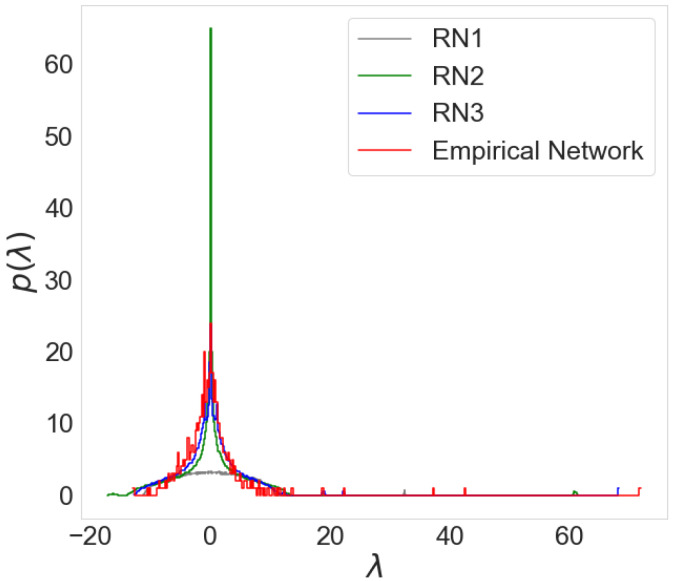
The probability distribution of eigenvalues for empirical network for year 2005 and three random networks.

Figure 3 shows the spectra of the empirical network obtained for 2005 and three random networks. The RN1 has the most different spectral properties than the empirical network, while the RN3 has the most similar spectra. Each random network only contains a fraction of information about the relations between nodes in the empirical network. The difference between spectra decreases as we increase the number of properties similar to the empirical network. Our analysis demonstrates that we can use the comparison between spectra to evaluate the optimal threshold.

We used the same approach to compare the full correlation matrix, represented as a weighted and filtered network.

The correlation matrix contains complete information about the system and can be represented as a weighted graph. Once the threshold is applied to the correlation matrix, edges with weights less than the threshold are removed. A filtered network thus only has a fraction of information about companies’ relations. By comparing the probability distributions of eigenvalues for original and filtered matrices, we understand how much information is lost due to filtering.

To quantify this difference, we use the Kolmogorov–Smirnov (KS) distance. We calculate the KS distance between the probability distributions of eigenvalues for the original and filtered correlation matrices for different threshold values. The lower value of KS distance implies a better agreement between spectra and higher similarity between networks’ topologies. Therefore, we want the KS distance to be as low as possible.

**Figure 4:**
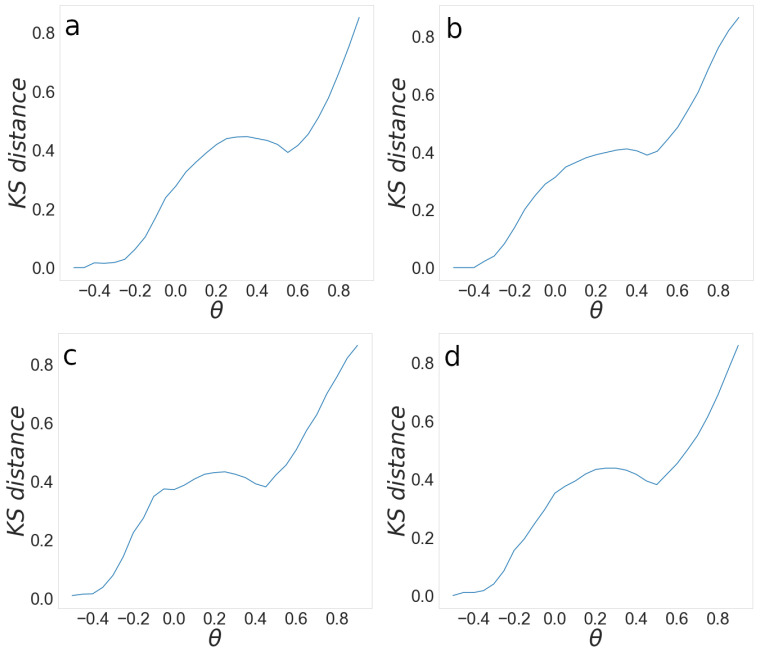
Kolmogorov–Smirnov distance between the probability distributions of the eigenvalues of correlation matrices obtained from original and filtered matrix for the years 2008 (**a**), 2009 (**b**), 2014 (**c**), and 2015 (**d**).

Figure 4 shows the KS dependence on the threshold for 2008, 2009, 2014, and 2015. As we expect, the KS distance increases with the threshold value. At the threshold −0.5, the KS distance is equal to zero as complete information is included in the network, while the KS distance reaches its maximum for a threshold close to 1. The dependence of KS on threshold has a local minimum at the value $\theta_{m}>0$ and is similar to the KS distance at the threshold θ=0. We keep the same information about the network structure by fixing the threshold’s value at 0 or $\theta_{m}$. However, a network at 0 is denser and thus more complicated for the analysis. By setting the threshold value to $\theta_{m}$ for which we observe the local minimum for the KS distance, and we obtain the optimal network with enough information about the relations between companies, which is not excessively dense.

**Figure 5:**
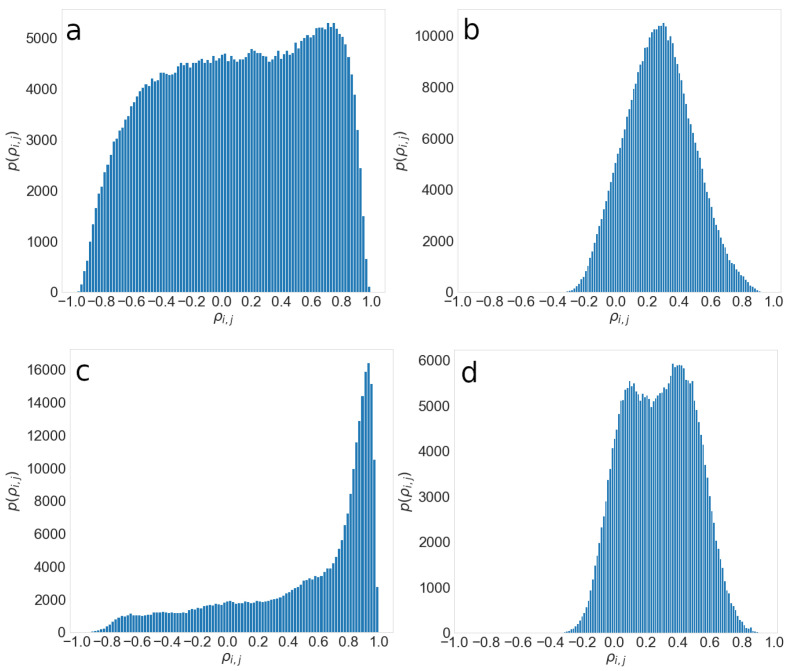
Probability distribution of the correlation coefficients for the years 2015, (**a**,**b**), and 2009, (**c**,**d**). (**a**,**c**) present distributions obtained from original time series, while (**b**,**d**) are the distributions obtained from detrended series.

The probability distributions of correlation coefficients differ for each year, as can be seen in Figure 5; thus, it is not surprising that the local minimum is different for each year. We calculate the local minimum for each year separately and obtain the network based on corresponding thresholds. Table 2 shows the local minima θ for different years.

#### 3.2.2. Measuring the Intra- and Inter-Community Connectivity

We are interested in the mesoscopic structure of the networks and how it changes with time. A community is a group of nodes more densely connected than the rest of the network [19]. Communities are an indicator of the system’s collective behavior, and the network’s community structure provides essential information about its dynamics and function [19]. In this work, we apply the Louvain algorithm [43] to find communities in weighted networks. The results of the Louvain algorithm for a single run may differ due to different initial conditions. We run a Louvain algorithm each year 100 times for these reasons. For each community $CM_{i}^{T,r}$, where *T* denotes a year and *r* denotes the run of the Louvain algorithm, we calculate the ratio between edges inside the community and all edges formed by nodes belonging to that community. We calculate the ratio using the following equation

(3) $$P_{in}^{CM_{i}^{T,r}}=\frac{L_{in}^{CM_{i}^{T,r}}}{L_{Total}^{CM_{i}^{T,r}}},\;i=1,2,...,R^{T,r}\;$$

where $L_{in}^{CM_{i}^{T,r}}$ is the sum of weighted edges inside the community $CM_{i}^{T,r}$, $L_{Total}^{CM_{i}^{T,r}}$ is the total sum of weighted edges of nodes in the community $CM_{i}^{T,r}$, and $R^{T,r}$ is the number of communities for the network obtained for time period *T* and run *r*. First, the average $P_{in}^{CM_{i}^{T,r}}$ over all communities obtained in the single run

(4) $$\langle P_{in}^{T,r}\rangle =\frac{\sum_{i}P_{in}^{CM_{i}^{T,r}}}{R^{T,r}}\;,$$

and then we obtain the average over all runs

(5) $$\langle P_{in}^{T}\rangle =\frac{\sum_{r}\langle P_{in}^{T,r}\rangle }{100}\;,$$

and standard deviation

(6) $$\sigma_{P_{in}^{T}}=\sqrt{\frac{\sum_{r}{\left(\langle P_{in}^{T}\rangle -\langle P_{in}^{T,r}\rangle \right)}^{2}}{99}}$$

## 4. Results

This work focuses on how the network structure changed when the system went through the 2008 economic crisis. We selected the period between 2002 and 2017, which covers the time before, during, and after the crisis. The number of companies varies between 518 in 2002 and 888 in 2017, as can be seen in Table 2.

We detrended each segment separately and calculated the correlation matrix $\left\{{\hat{\rho }}_{i,j}\right\}$ between the companies for each year T∈{2002,...,2017}. We detrended the time series for the interval l=21 trading days, which equals one average trading month. We then mapped the correlation matrix to the adjacency matrix using the threshold method and obtained an undirected weighted network for the year *T*. We use the approach described in Section 3.2 to determine the threshold. We performed community structure analysis and calculated the $\bar{P_{in}^{T}}$ and $\sigma_{P_{in}^{T}}$ for each year. The analysis of community structure and the evolution of their cohesion shows how the network structure evolves.

### 4.1. Characteristics of the Correlation Matrix and Obtained Network

Detrending helps extract information about the economic system’s internal behavior and relationships between companies. Figure 5 shows a probability distribution of correlation coefficients $p({\hat{\rho }}_{i,j})$ for the original time series and detrended time series for years 2009 and 2015. The $p_{ij}\left({\hat{\rho }}_{i,j}\right)$ shown in Figure 5a was calculated for the original time series for the year 2015 and resembles a uniform distribution. Figure 5b shows the probability distribution obtained from the detrended series and is more similar to Gaussian distribution. The center of Gaussian varies between years. The distribution of correlation coefficients changes during the economic crisis period, as can be seen in Figure 5c,d. If we obtain the correlation matrix from the original time series, most companies are highly correlated, with correlation coefficients between 0.9 and 1, as can be seen in Figure 5c. The distribution of correlation coefficients obtained from detrended time series during an economic crisis is a convolution of two Gaussians, Figure 5d.

**Figure 6:**
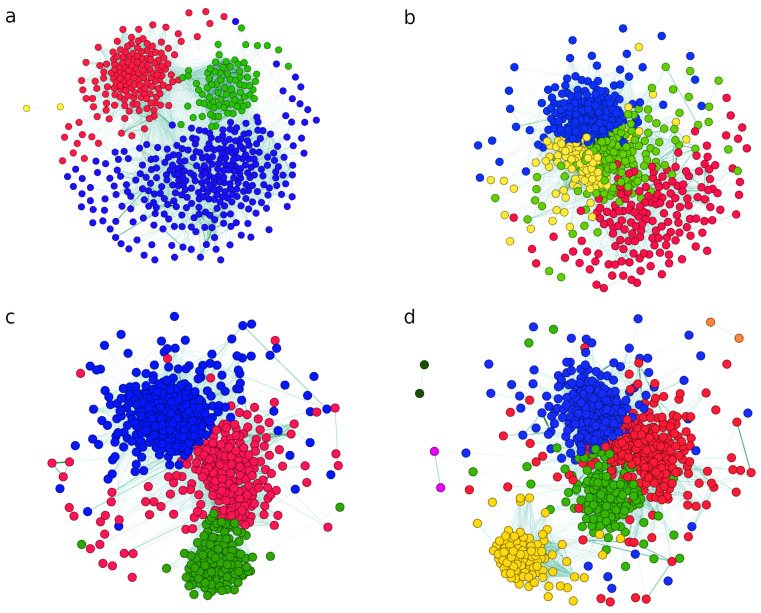
Networks obtained from detrended time series in the years 2004 (**a**), 2006 (**b**), 2008 (**c**), and 2015 (**d**). Networks are obtained by applying the threshold given in Table 1. The number of nodes and edges, respectively, is equal to 554 and 23,340 (**a**), 652 and 43,167 (**b**), 677 and 47,590 (**c**), and 793 and 58,291 (**d**). Nodes of the same color belong to the same community.

After detrending the time series and calculating the correlation matrix, we used a method described in Section 3.2 to obtain an undirected weighted network. We ran Louvain on the networks and found the community structure. Figure 6 shows the networks for the years 2004, 2006, 2008, and 2015. Based on examining communities by comparing their constituents’ characteristics, we concluded that their edges imply exposure to similar factors. Namely, the nodes belonging to a community, i.e., companies in the same sector, have different owners, operate in different states, and have different clients. Common to these companies is their economic activity, i.e., their functioning is similar. Therefore, we obtain a network where edges reflect exposure to similar factors.

Inter-community edges indicate that even companies belonging to different subsectors may operate under similar conditions. For example, a bank and REIT company are exposed to similar external factors if they are linked to the residential project, where a bank lends money to home buyers while REIT invests in project development.

Robust intra-community connectivity indicates that companies in the same community operate under similar conditions and are susceptible to the same factors. The low value of correlation coefficients between companies belonging to different sub-sectors suggests that specific factors typically affect them. Strong connectivity between network nodes is an indicator of its high vulnerability. A system with a distinct community structure and stronger connectivity within the communities than between the communities is more robust than one with similar strengths of connections between and within the communities.

We are interested in the evolution of the ratio between intra- and inter-community connectivity and how this ratio changes when the system is in different states, such as during crisis and out-of-crisis periods.

### 4.2. Relation between Inter and Intra-Connectivity of Communities and Its Evolution

**Figure 7:**
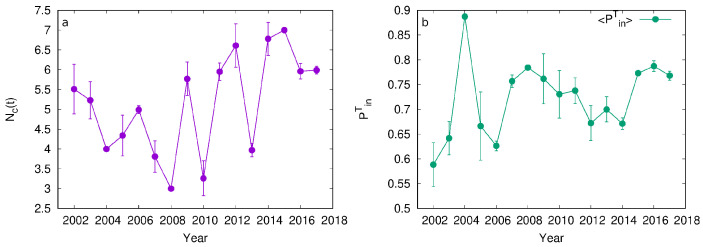
The evolution of the average number of communities (**a**) and average intra-connectivity (**b**) for networks from 2002 to 2017.

We analyze the community structure of networks for each year from 2002 to 2017 using the Louvain method. The results of applying the Louvain method, which includes the number and structure of communities, depend on the initial conditions. As a result, different runs of the Louvain algorithm on the same network may result in a different number of communities depending on how network nodes are assigned to these communities. For these reasons, we ran the Louvain algorithm 100 times on each of the 16 networks and calculated the average number of communities and the average connectivity of these communities. Figure 7a shows the evolution of the number of communities between 2002 and 2017. The number of communities fluctuates with time and grows after the peak of the crisis in 2008, with two distinctive local minima in 2010 and 2013. Furthermore, the number of detected communities in 2004, 2008, and 2015 is equal for each of the 100 runs of the Louvain algorithm, suggesting a stable community structure in these networks. We observe the lowest number of communities for 2008, which indicates the lowest differentiation between sectors within the financial industry during the financial crash.

We analyze the intra- and inter-community connectivity for the networks obtained for each year from 2002 to 2017. Figure 7b shows <$P_{in}^{T}$> for the years from 2002 to 2017. Higher values of <$P_{in}^{T}$> imply higher community intra-connectivity, while lower values indicate higher community inter-connectivity. The error bars shown in Figure 7b are standard deviations calculated on the sample of 100 runs. Low standard deviation implies similar intra-community connectivity among communities. The peak of intra-community connectivity is observed in the year 2004. The interconnectivity then drops to its minimal value in 2006, where the connectivity within the communities grows and has local maxima in 2008, which slowly decreases until 2014. In 2015, we observed another smaller rise in connectivity.

The networks of the years 2004, 2008, and 2015 have two essential features. They have a very stable number of communities, independent from the initial conditions of the Louvain method. Furthermore, the intra-community connectivity for these networks has a local maximum in these years with a very low standard deviation.

Figure 7a shows that the intra-community connectivity $\langle P_{in}^{T}\rangle$ has the local minima in the year 2006, indicating high connectivity between communities. In 2006, the system had the highest potential for diffusion between communities, meaning that one community’s disturbance could easily be transmitted to any other community. If this disturbance is a failure, the system is at high risk of efficiently spreading failure and breaking down. Our result matches what happened to the USA financial sector since 2006 was the year before the crisis started in 2008. Other researchers have predicted the beginning of the crisis [1]. High and consistent inter-community connectivity in 2006 indicates that companies in different sectors were susceptible to the influence of the same factor. This factor was real estate lending, which pulled most of the financial industry. Many financial sectors were directly or indirectly involved in real estate lending, leading to the relationship network’s almost homogeneous structure. The local minima in 2006 preceded a peak in 2004, where communities were well defined.

**Figure 8:**
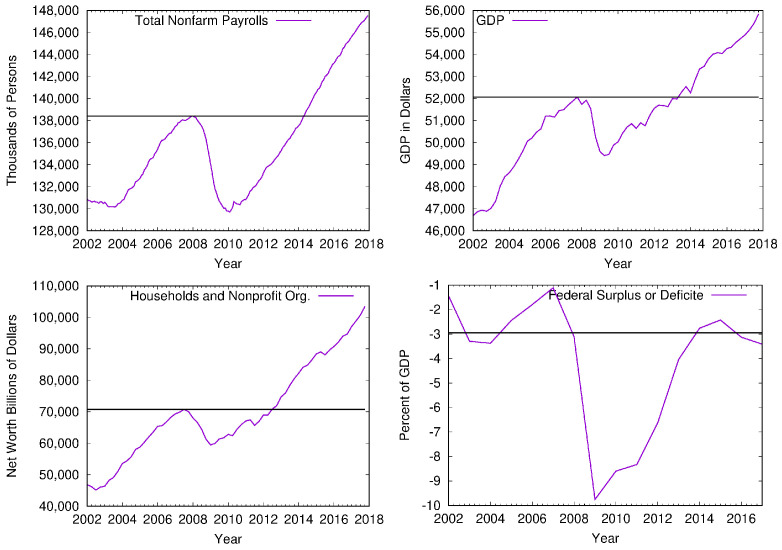
Economic variables which indicate whether the system is in the state of crisis or out of crisis such as (left top) employment, (right top) gross domestic product, (left bottom) household net worth, and (right bottom) federal surplus or deficit.

A crisis is followed by a period of recession, which is recognized by lower values of economic indicators such as employment, gross domestic product, household net worth, and federal surplus or deficit. Figure 8 shows the relative change of these four indicators for the USA economy between 2002 and 2017. We see that the recession period lasted from 2009 and ended in 2014. Our results indicate that the standard deviation for intra-community connectivity has higher values for the same period, while its values decrease between 2014 and 2017. We see from Figure 7b that standard deviation $\langle P_{in}^{T}\rangle$ was higher during the economic recovery compared to the post-crisis period.

We observe the increase in $\langle P_{in}^{T}\rangle$, see Figure 7a in the years 2007 and 2008, after reaching its minimum in 2006. In 2007, companies in the financial system understood that the economy was in bad condition and that interconnection was high. Communities tried to depart from each other, leading to a high $\langle P_{in}^{T}\rangle$ and low $\sigma_{P_{in}^{T}}$ in the year 2008. However, the number of communities $N_{C}\left(T\right)$ decreased in 2008 because two communities merged into one, regional banks and REITs. We observed the homogenizing of the system in a different form where the number of different sectors decreased.

#### Effect of Regulation on Structure and Behavior

The USA financial system has to be controlled to prevent the system break down and decrease systemic risk [1]. Control is realized through appropriate regulations. High restrictive regulation may prevent a crisis. However, it can jeopardize economic growth since it limits companies’ profits [1]. Less stringent regulations enable higher yields but increase systemic risk. Therefore, an optimal level of regulation has to be implemented, allowing a thriving economy while decreasing systemic risk. The regulations impose restrictions on companies’ behavior, while deregulation provides companies with a higher degree of freedom. They both define the behavior of comprising elements of the system. The effect of regulation and deregulation on the system results from the collective behavior of incorporating elements. One needs tools to measure the impact of regulations on the system to create optimal regulations. Our methodology provides insight into the influence of regulation and deregulation on a system’s structure and behavior.

Deregulation took place in 2004 [44] and proposed a system of voluntary regulations where investment banks can hold less capital in reserve. Having less money in reserve means that companies become more dependent on other companies and more vulnerable. Higher connectivity between companies leads to an increase in systemic risk. Deregulation is considered one of the leading causes of crisis [45]. Our results show that $\langle P_{in}^{T}\rangle$ sharply decreased in 2005, indicating higher inter-dependence between communities and higher systemic risk. $\langle P_{in}^{T}\rangle$ and the standard deviation $\sigma_{P_{in}^{T}}$ further decreased in 2006, implying higher homogeneity within the system.

Regulations were implemented between 2011 and 2014 to respond to the crisis. The Dodd–Frank Wall Street Reform and Consumer Protection Act of 2010 was designed to increase financial stability and prevent future crises [46]. As this was the most comprehensive overhaul of the financial system [47], it took time to be implemented. Implementation started in 2011 and reached 50% of planned regulations in 2014 [48]. Our results show a sharp increase in $\langle P_{in}^{T}\rangle$ in 2014 when the economy recovered. Standard deviation $\sigma_{P_{in}^{T}}$ is higher during the crisis period compared to the period of the recovering economy, 2014–2017.

## 5. Discussion and Conclusions

In this work, we used a novel method to infer network structure from time series to study the cohesion between USA companies in the financial sector. Compared to existing methods, we used detrended prices instead of detrended returns. We introduced a technique for obtaining an optimal network from a correlation matrix and used a measure based on community structure that allows us to examine the evolution of cohesion. Our results show that the USA financial system’s network structure between 2002 and 2017 underwent several phases: deregulation, crisis, and post-crisis. Each of these periods is characterized by different intra-community connectivity and standard deviation. The strength of connections between communities is directly related to the system’s level of risk and stability.

Understanding the connections between the system’s components is crucial for preventing crises. Our approach can identify the points of high systemic risk. This knowledge enables timely actions to increase the system’s stability. Moreover, measuring the effect of these actions, such as regulation and deregulation, can be performed using our method. This is of great importance as inadequate efforts can further deteriorate financial stability. In 2008, the government’s actions to increase financial stability and save the economy in the form of capital injection into the financial system were inadequate, which further pushed the economy into recession [1]. The price of wrong measures for recovering the economy is high in times of crisis because resources are even more limited. Our results show that the system’s structure did not change due to these measures.

The economic system has to be regulated to prevent crises while securing the unrestrained behavior of individual companies to allow economic growth and prosperity. The economic system is dynamic and should be constantly monitored by policymakers to secure an optimal trade-off between of the economic growth and limiting behavior of composing elements. Policymakers must act on time since a delay of adequate actions can have a negative impact. Our method allows policymakers to see whether their actions are adequate and act promptly. As per our analysis, deregulation, which took place in 2004 to enable economic growth, had a strong impact on increasing systemic risk. This signal can be seen in 2005, where $\langle P_{i}n\rangle$ sharply decreased, while standard deviation implied that connectivity between a certain number of communities increased. In addition, in 2006, all communities were strongly interconnected, which presents a high systemic risk and can be seen in low $\langle P_{in}\rangle$, standard deviation, and the number of communities. This led to the 2008 crisis, when some of the communities merged.

Existing techniques for constructing networks from the correlation matrix, MST and PMFG, put strict constraints on the network structure. MST forbids cycles between nodes and conditions the number of links to N−1, where *N* is the number of nodes. PMFG only allows short cycles and the maximal number of links 3(N−2). There is no economic reason behind these topological constraints for economic systems. Furthermore, the limit on the number of connections is too strict and may filter out some critical information about the network’s connectivity. The lack of this forbids the study of the cohesion of the network and its dynamics.

Our method can be used by researchers interested in studying collective behavior in real systems such as economic, social, biological, and technological systems. The pre-requisite is the availability of data in the form of time series. Our method enables discovering hidden relationships between the constituents of the system, leading to a better understanding of the system, predicting its behavior and controlling it.

## Figures and Tables

**Table 1:** The number of USA financial sector companies in each year *T* according to the Yahoo database.

*T*	$N_{c}\left(T\right)$	*T*	$N_{c}\left(T\right)$
2002	518	2010	762
2003	558	2011	786
2004	609	2012	804
2005	653	2013	825
2006	695	2014	855
2007	711	2015	884
2008	740	2016	892
2009	748	2017	888

**Table 2:** The threshold values obtained for different years.

Year	θ	Year	θ
2002	0.35	2010	0.525
2003	0.325	2011	0.55
2004	0.425	2012	0.475
2005	0.35	2013	0.475
2006	0.35	2014	0.45
2007	0.475	2015	0.5
2008	0.55	2016	0.55
2009	0.475	2017	0.375

## Data Availability

The data used in this research are publicly available and can be found at https://figshare.com/articles/dataset/Financial_data/20088311 accessed on 17 June 2022.
